# Reported patterns of vaping to support long-term abstinence from smoking: a cross-sectional survey of a convenience sample of vapers

**DOI:** 10.1186/s12954-020-00418-8

**Published:** 2020-10-06

**Authors:** Sarah Victoria Gentry, Emma Ward, Lynne Dawkins, Richard Holland, Caitlin Notley

**Affiliations:** 1 Norwich Medical School, University of East Anglia, Norwich Research Park, Norwich, NR4 7TJ UK; 2 Centre for Addictive Behaviours Research, School of Applied Sciences, London South Bank University, 103 Borough Road, London, SE1 0AA UK; 3 George Davies Centre, Leicester Medical School, University of Leicester, Lancaster Road, Leicester, LE1 7RH UK

**Keywords:** e-Cigarettes, Smoking relapse, Cross-sectional survey

## Abstract

**Background:**

E-cigarettes are the most popular aid to smoking cessation attempts in England and the USA. This research examined associations between e-cigarette device characteristics and patterns of use, tobacco-smoking relapse, and smoking abstinence.

**Methods:**

A convenience sample of 371 participants with experience of vaping, and tobacco-smoking abstinence and/or relapse completed an online cross-sectional survey about e-cigarettes. Factors associated with smoking relapse were examined using multiple linear and logistic regression models.

**Results:**

Most participants were self-reported long-term abstinent smokers (86.3%) intending to continue vaping. Most initiated e-cigarette use with a vape pen (45.8%) or cig-a-like (38.7%) before moving onto a tank device (89%). Due to missing data, managed through pairwise deletion, only around 70 participants were included in some of the main analyses. Those using a tank or vape pen appeared less likely to relapse than those using a cig-a-like (tank vs. cig-a-like OR = 0.06, 95% CI 0.01–0.64, *p* = 0.019). There was an inverse association between starting self-reported e-cigarette liquid nicotine concentration and relapse, interacting with device type (OR = 0.79, 95% CI 0.63–0.99, *p* = 0.047), suggesting that risk of relapse may have been greater if starting with a low e-cigarette liquid nicotine concentration and/or cig-a-like device. Participants reported moving from tobacco-flavored cig-a-likes to fruit/sweet/food flavors with tank devices.

**Conclusions:**

Knowledge of how people have successfully maintained tobacco-smoking abstinence using vaping could help other tobacco smokers wishing to quit tobacco smoking through vaping.

## Background

E-cigarette use, known as “vaping,” is thought to be less harmful than tobacco smoking [1] and e-cigarettes are the most popular aid to smoking cessation attempts in England [2] and the USA [3]. Estimated current e-cigarette use prevalence among tobacco smokers in the UK is 21.9%, and 36.5% report ‘ever use’ [4]. In the USA, 15.9% report current use and 47.6% ever use [5]. Regular (at least weekly) e-cigarette use among never smokers in Great Britain has been very rare (< 1%) [4] and past week vaping by never-smoking adolescents in the USA was 3% in a 2018 survey [6].

**Fig. 1:**
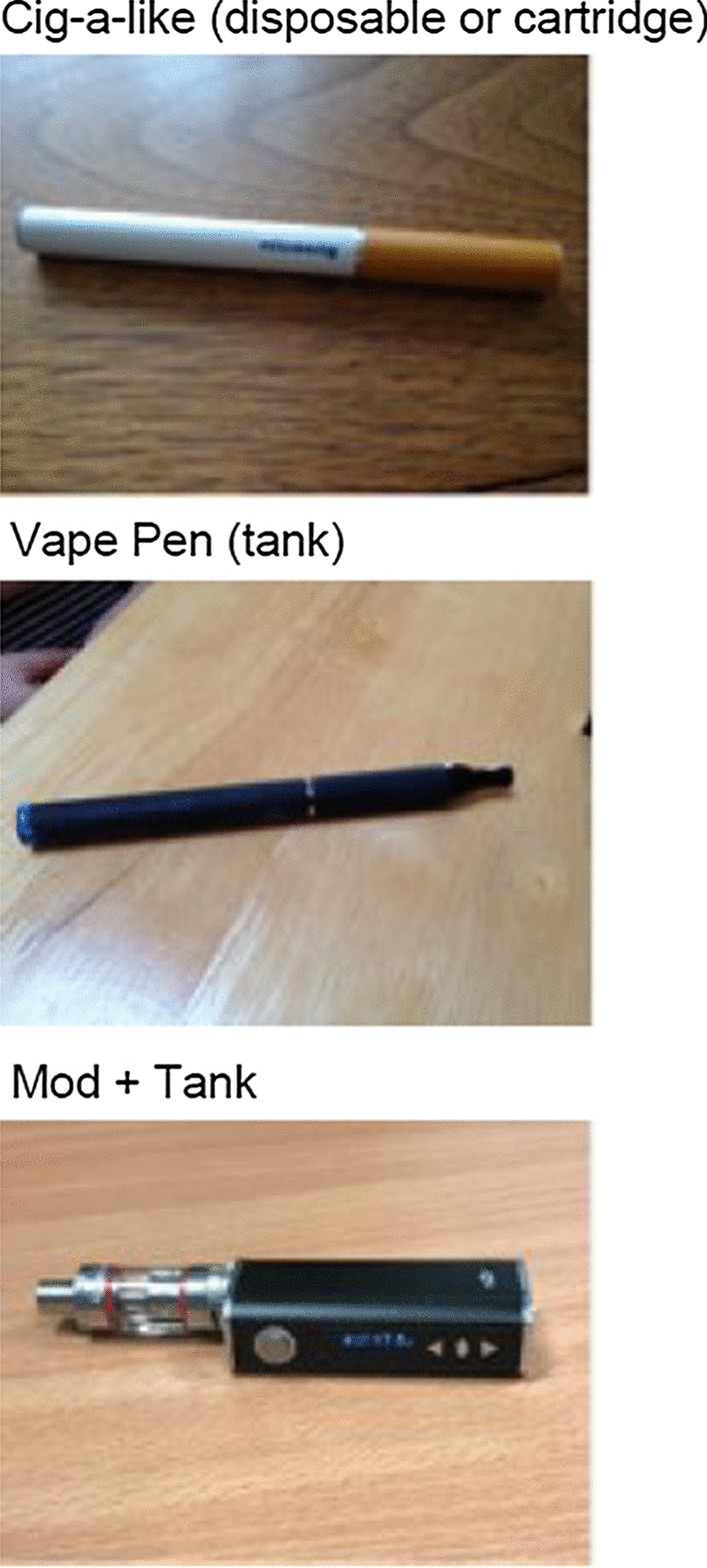
Examples of first-, second-, and third-generation e-cigarettes

E-cigarettes are electronic devices that heat “e-liquid” (usually comprised of propylene glycol and glycerol, with or without nicotine and flavors) stored in a disposable/refillable cartridge/reservoir to form an aerosol for inhalation [7]. E-cigarettes are commonly referred to as being first, second, or third generations (Fig. 1). First-generation devices are typically “cig-a-likes” designed to look and feel like tobacco cigarettes and use prefilled cartridges [8]. Second-generation devices generally appear like pens, use tanks that can be re-filled and have larger battery capacity. Third-generation devices use re-fillable tanks and allow modifications to the voltage and/or wattage output, improving performance and allowing a tailored user experience. Pod devices, designed to combine the simplicity of cig-a-likes with the user experience of third-generation devices, were released onto the market in the USA in 2015 and subsequently became available in the UK [4].

Use of e-cigarettes has grown rapidly and may support smoking cessation, but there is little evidence on long-term health effects or sustained smoking cessation. A Cochrane review [7] identified two randomized controlled trials (RCTs) [9, 10] suggesting e-cigarettes are more effective for smoking cessation compared with placebo e-cigarettes and one RCT found no significant differences between e-cigarettes and nicotine patch. However, overall evidence was low quality. Two large RCTs have been published since completion of this Cochrane review. An RCT comparing provision of free cartridge e-cigarettes with low nicotine delivery, compared with nicotine patches, suggested a similar low efficacy for both treatments (1% and 0.5% sustained abstinence at 6 months respectively) [11]. An RCT comparing a group assigned to an e-cigarette starter pack (second-generation refillable e-cigarette, one bottle of 18 mg e-liquid and recommendations to purchase further liquid of their choice) and a group assigned to nicotine replacement therapy (NRT) of their choice, both with associated behavioral support, found 18.8% 1 year biochemically verified smoking abstinence in the e-cigarette group compared with 9.9% in the NRT group (RR 1.83; 95% CI 1.30–2.58, *p* < 0.001) [12].

Most attempts at smoking cessation result in relapse, and smokers generally make multiple quit attempts before succeeding [13]. Qualitative research suggests e-cigarettes can meet many of the needs of ex-smokers by substituting physical, psychological, social, cultural and identity-related aspects of tobacco addiction [14, 15]. According to a time-series analysis of data from the Smoking Toolkit study, in which repeated cross-sectional surveys are conducted with a representative sample of households in England, increasing prevalence of e-cigarette use in current smokers was predictive of higher success rates of quit attempts [16].

There is evidence that how people use e-cigarettes, in terms of device type and patterns of use, can affect number and success rate of quit attempts. Brose et al. found that daily, but not non-daily, e-cigarette use is predictive of greater cigarette cessation attempts and reduced smoking among UK adults [17]. Evidence suggests abstinence from smoking may be significantly higher among tank users [18]. Another determining factor in e-cigarette effectiveness is the nicotine concentration used. The amount of nicotine delivered varies depending on a range of characteristics, including the device (e.g., model, wattage), e-liquid (e.g., flavor, ingredients, pH) and user behaviour (e.g., puffing topography) [19, 20]. A Cochrane review of NRT for smoking cessation suggested heavier smokers required higher nicotine doses [21]. According to an ethnographic study, vape shops recommend higher e-liquid nicotine concentrations for heavier smokers [22], but this is not yet backed up by robust evidence.

Current understanding of how e-cigarette users, known as “vapers,” use e-cigarettes to avoid smoking relapse, is limited. This study reports results of an online survey from a convenience sample of vapers to elucidate patterns of use and types of devices that might best support ongoing tobacco-smoking abstinence. Hypotheses were generated based on the associated qualitative study [15]. We hypothesized:

1. Those who initiate vaping using a first-generation device are more likely to relapse to tobacco smoking than those initiating using a later-generation device;
2. Those who start on a low self-reported nicotine e-liquid concentration (strength) will be more likely to relapse to tobacco smoking than those starting on a higher nicotine e-liquid, after controlling for cigarettes per day (CPD) before cessation;
3. There will be a relationship between lower nicotine strength, interacting with device type, and relapse, as newer-generation devices provide nicotine more efficiently [23].

## Methods

### Participants

A convenience sample of UK vapers were invited to participate in an online survey, combining fixed choice and open-ended responses, collecting quantitative and qualitative data. This paper reports analyses of the quantitative data collected.

Participants who self-reported a history of tobacco smoking, experience of using an e-cigarette and tobacco-smoking abstinence or relapse following e-cigarette use, were initially recruited to participate in a qualitative study [15, 24]. When the qualitative study reached “saturation,” further volunteers were diverted to the survey. Recruitment was through word of mouth, press articles, university bulletins, and social media.

### Procedure

Informed consent was obtained. Question items were designed based on topics from the accompanying qualitative study. Questions were asked on socio-demographic characteristics, tobacco variables, e-cigarettes and previous quit attempts. Data on relapse were obtained by asking participants whether they were abstinent from smoking after using their first device (“yes” or “no, I relapsed”). For data on e-cigarette device use, we asked “What type of e-cigarette device did you try first? Please select the picture that looks most like the device you started with”, “Did your first device help you stay stopped from smoking tobacco?” and “What type of e-cigarette device do you currently use the most?”.

There were two versions of the survey which were combined for analysis. One of the questions from Version 1 (V1 *n* = 183) on devices used was poorly completed and complex and was re-designed for Version 2 (V2 *n* = 188). V1 asked participants to list devices they have used in the order they used them, starting with the first one tried to the one currently used. Free-text boxes were provided for the device name, wattage, nicotine strength (e.g., 12 mg, 6 mg) and flavorings (e.g., tobacco, fruit). V2 asked participants to select the type of device they tried first and the device used currently in a multiple-choice question including cig-a-like, vape pen, mod and tank, and other. Options were accompanied by an example photo. They were then asked to detail in free text boxes their current and starting flavor (e.g., tobacco, fruit) and nicotine strength. Free text responses on device type from V1 were coded as per the categories for V2 and included in this analysis. Data described below includes participants from both versions.

The study received ethical approval from the University of East Anglia Faculty of Medicine and Health Sciences Research Ethics Committee.

### Analysis

Participant characteristics, tobacco and e-cigarette variables were examined with descriptive statistics. Binary logistic regression was used to examine associations between device type or nicotine strength and current/previous self-reported relapse. Relapse was defined as “a successful smoking quit attempt of at least 48 h, followed by a relapse (more than five instances of smoking) to tobacco smoking.” This was chosen in order to capture both early and late relapsers, whilst excluding dual users and triallers (those who use e-cigarettes alongside tobacco smoking without making a serious quit attempt). Five episodes were required for relapse as per the Russell Standards which allow up to five cigarettes to be smoked for an individual still to be considered smoking abstinent [25]. After examining these associations, the interaction between device type and strength and its possible association with relapse was investigated by adding an interaction term for strength and device to the model.

Multiple linear regression was used to examine associations between CPD and e-liquid nicotine starting strength.

Missing data were dealt with through pairwise deletion in order to increase power. Where percentages are reported in the results section, this is the percentage of those for whom there were data available for that variable. Here “*n*” is used in the results section this is the absolute number of participants who gave that response, so where there is missing data this will not total 371.

All models were built up stepwise, controlling for age and sex, followed by CPD.

In the results section, descriptive statistics are presented, followed by the results for each of the three hypotheses tested.

## Results

### Descriptive statistics

**Fig. 2:**
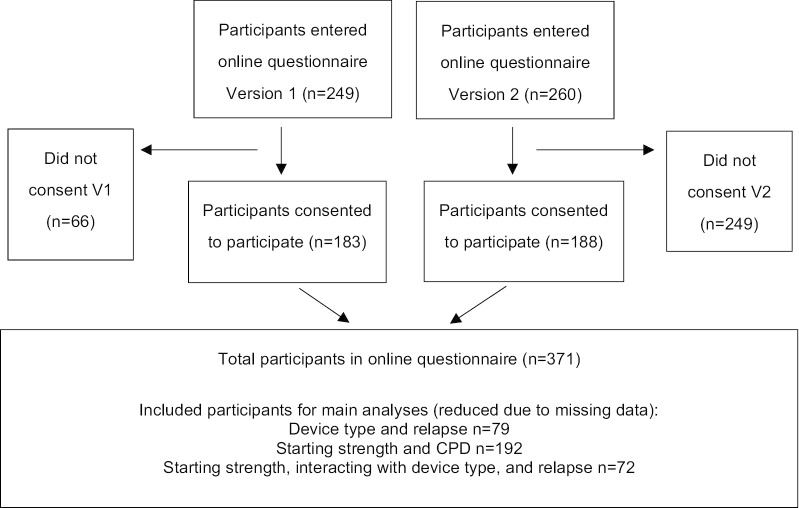
Survey participant flow diagram

509 participants entered the online survey (V1 *n* = 249, V2 *n* = 260). Of these, 27.1% did not give consent to participate and were discarded (V1 *n* = 66, V2 *n* = 72), leaving 371 participants (V1 *n* = 183; V2 *n* = 188). Of these 371 participants’ the number of responses to individual questions varied. Questions on flavors, device types, e-liquid nicotine strengths and vaping status were answered by more than 60% of the 371 participants. Questions on smoking status were answered by 56.9% of participants. Questions on relapse, the main outcome variable, were only answered by 42% of participants. Sample sizes for key outcomes are presented in Fig. 2.

#### Demographic characteristics

23.6% of participants were female and mean age was 49 (SD 11.61, range 22–78). Participants came from across the UK. Eight participants (< 1%) reported living outside of the UK.

Half of participants (50.0%) were married (*n* = 108/216), 26.4% were single (*n* = 57/216), 14.8% were cohabiting (*n* = 32/216), 6.0% were divorced (*n* = 13/216) and 2.7% were widowed (*n* = 6/216).

92.3% (*n* = 84/91) described themselves as “White”, 10.7% (*n* = 9/91) of whom specified “White European”. The remainder were “Mixed” (*n* = 6/91) or “Asian” (*n* = 1/91).

Regarding occupation, 24.1% (*n* = 39/162) described themselves as “retired” and 6.2% (*n* = 10/162) “self-employed”. The remaining 69.7% of participants were in work (*n* = 113/162) and were classified according to the NRS Social Grade classification. Of those in work, 19.1% (*n* = 22/113) were social grades A and B, 47.6% (*n* = 53/113) social grades C1 and C2 and 33.3% (*n* = 38/113) grades D and E.

#### Smoking and vaping status

Most participants were long-term abstinent from tobacco smoking (defined as ≤ 5 instances of one-off tobacco-smoking relapse in the last 12 months) (86.3%, *n* = 182/211), > 99% of whom continued to vape. A further 10.4% had recently quit smoking and were vaping (*n* = 22/211) and 3.1% (*n* = 7/211) were vaping and occasionally smoking. Most intended to continue vaping (85.4%, *n* = 194/227). < 1% had already stopped using e-cigarettes.

Of those still using e-cigarettes (*n* = 256), the average reported duration of use was 3.9 years (SD 2.3, range 1 month to 9.8 years).

#### Self-reported e-liquid nicotine concentration (strength) used

Mean initial nicotine strength was 19.84 mg (SD 8.97, range 0–48 mg), reducing to an average 7.96 mg with their current device (SD 7.06, range 0–36 mg). Higher mean CPD generally appeared to coincide with higher starting strength, except in the group starting with 0-6 mg nicotine e-liquid (Table 1).

**Table 1:** Starting e-liquid nicotine strengths and cigarettes per day

Starting strength (mg)	Participants (*n* = 200)	Mean CPD
0–6^a^	21	28.3
7–12	25	23.8
13–18	67	28.2
19–24	57	30.5
25+	30	36.8

^a^Note only a single participant reported using 0 mg/ml as a starting e-liquid nicotine concentration

#### Self-reported e-liquid flavors used

Results suggest a change in flavor choices over the course of vaping initiation and uptake. There was a reduction in the proportion of people using a tobacco flavor (− 36.5%, 95% CI − 43.5 to − 29.6), and increase in the proportion using a fruit/sweet/food flavor (+ 31.7%, 95% CI 23.3–40.0%), from initial to current flavor choice (Additional file 1: Table A1).

#### Self-reported devices used

Most participants reported their initial device was either a vape pen (second generation) (45.8%) or cig-a-like (first generation) (38.7%). Most reported their current device to be a mod and tank (third generation) (89.4%).

No differences in demographic characteristics of those who chose different initial devices were identified (Additional file 1: Table A2).

**Table 2:** Device types and relapse

Device type	Initial device type (*n* = 238)	Relapse with initial device (*n* = 98 asked and responded to this question)	Final device type (*n* = 235)
Cig-a-like	92 (38.66%)^a^	22/42 (52.38%)	3 (1.28%)
Vape pen	109 (45.80%)	6/40 (15%)	22 (9.36%)
Mod and tank	38 (16.00%)	1/16 (6.25%)	210 (89.36%)
All	238	29/98 (29%)	235

^a^Note percentages do not total exactly 100 due to rounding

Among those who became successfully abstinent from smoking using their first device (44.9%, *n* = 71), 71.8% (*n* = 51) switched to another device, all of whom moved from an earlier to later-generation device. Of those who became successfully abstinent from smoking using their first e-cigarette device and continued to use the same or a similar type of device (*n* = 20), 65.0% (*n* = 13) had started with a mod and tank device, 24.0% (*n* = 5) a vape pen and only two successfully stopped smoking with, and continued to use, a cig-a-like.

Coded self-reported reasons as to why participants moved on from their first device indicate that most wished to upgrade to a device they perceived as better (*n* = 57) or to move on from a device they considered inadequate for their needs (*n* = 21) due to battery life/power, flavor and improved technology. Some wished to upgrade to enjoy opportunities to personalize their device or as a hobby (Additional file 1: Table A3). Mean number of devices tried was 5.4 (SD 2.96, range 1–15, *n* = 98).

**Table 3:** Comparison of participant demographics with the Smoking Toolkit Study

	Smoking Toolkit Study	This study (ECtra)
Mean age	39.5 (SD 15.6)	49 (SD 11.61)
Female (%)	54	23.63
*Social grade (% in each category)*		
A	10.7 (*A* + *B*)	10.5 (*n* = 17)
B		8.6 (*n* = 14)
C1	22.9	38.3 (*n* = 62)
C2	22.7	9.3 (*n* = 15)
D	18.8	15.4 (*n* = 25)
E	24.8	17.9 (*n* = 29)

### Results for hypothesis 1

#### **Hypothesis 1**

Those who initiate vaping using a first-generation device are more likely to relapse to tobacco smoking than those initiating using a later-generation device.

Only 44.9% (*n* = 71/158) reported successful tobacco abstinence after using their first device. 24.7% (*n* = 39/158) reported dual use, 18.4% (*n* = 29/158) full relapse and 12.0% (*n* = 19/158) occasional lapses. Relapse appeared more common among those using a cig-a-like, compared with a vape pen or a mod and tank (Table 2).

Those using a mod and tank device (*n* = 16/98), or a vape pen (*n* = 40/98), on initiation were significantly less likely to relapse than those using a cig-a-like (*n* = 42/98) (mod and tank vs. cig-a-like OR = 0.06, 95% CI 0.01–0.50, *p* = 0.009; vape pen vs. cig-a-like OR = 0.16, 95% CI 0.06–0.46, *p* = 0.001, *n* = 98). This difference remained after controlling for age and sex (mod and tank vs. cig-a-like OR = 0.06, 95% CI 0.01–0.64, *p* = 0.019; vape pen vs. cig-a-like OR = 0.14, 95% CI 0.04–0.51, *p* = 0.003, *n* = 79).

### Results for hypothesis 2

#### **Hypothesis 2**

Those who start on a low self-reported nicotine e-liquid concentration (strength) will be more likely to relapse to tobacco smoking than those starting on a higher nicotine e-liquid, after controlling for cigarettes per day (CPD) before cessation.

Mean initial nicotine strength was 19.84 mg (SD 8.97, range 0–48 mg) and mean current nicotine strength was 7.96 mg (SD 7.06, range 0–36 mg), suggesting most participants reduced nicotine strength over time.

Splitting data on initial e-liquid nicotine strengths into quartiles suggested those with higher reported CPD may use higher starting strengths. A simple linear regression was calculated to predict starting nicotine strength based on CPD. A significant association was found, with nicotine strength increasing by 0.1 mg for every extra cigarette smoked per day (95% CI 0.025–0.187, *p* = 0.01, *n* = 200). The strength of the association reduced slightly but remained statistically significant after controlling for age and sex (0.08 mg increase, 95% CI 0.003–0.164, *p* = 0.041, *n* = 192).

There was no association between initial self-reported e-liquid nicotine content and relapse (OR = 1.00, 95% CI 0.95–1.05, *p* = 0.895, *n* = 100) and there continued to be no association after controlling for age, sex, device type and CPD (OR = 1.01, 95% CI 0.95–1.09, *p* = 0.677, *n* = 72).

### Results for hypothesis 3

#### **Hypothesis 3**

There will be a relationship between lower nicotine strength, interacting with device type, and relapse, as newer-generation devices provide nicotine more efficiently [20].

After adding an interaction term for initial strength and device type, there was a small but statistically significant inverse association between starting strength and relapse (OR = 0.79, 95% CI 0.63–0.99, *p* = 0.047, *n* = 72). There was a significant interaction term for the device type and nicotine strength interaction (OR = 1.18, 95% CI 1.02–1.37, *p* = 0.026, *n* = 72).

## Discussion

This study of real-world patterns of e-cigarette use suggests choice of products and liquids may impact tobacco-smoking relapse.

Relapse was much more likely among people initiating e-cigarette use with a cig-a-like, compared with other types of device. According to an ethnographic study, vape shops sometimes separate devices into “beginner,” “intermediate,” and “advanced” in displays [22]. Cross-sectional surveys by McNeill et al. suggested newer e-cigarette designs were more effective for smoking cessation (38.0%) than older ones(19.9%) [18, 26]. A study of 50 smokers unwilling to quit who were provided with second-generation e-cigarettes found 36% CO verified smoking abstinence after 24 weeks [27]. An online survey by Etter suggested that users perceived tank devices as more effective than pre-filled models for smoking cessation [28]. Our study supports these findings but classified devices into three categories (cig-a-like, vape pen and tank), providing additional granularity. As our data is cross-sectional, it is unclear whether progressing from a simpler device to a more complex one is beneficial, such as allowing the user to develop skills in adapting their device to suit their needs, or if new users should be recommended to start with a newer device.

Most shops in an ethnographic study of vape shops used “rules of thumb” when recommending nicotine strengths to customers [22]. Smokers of ≤ 10 CPD are recommended 3–6 mg nicotine, 10–20 CPD 6–12 mg and 20+ CPD 18 mg. Our results suggest that in practice vapers who reported higher CPD generally start with higher nicotine strength e-liquids, but that perhaps some smokers are starting on strengths insufficient for their needs. Mean CPD for those starting on 0–6 mg nicotine was 28.3, much higher than the 10 CPD advised by vape shops (Table 2). Mean initial nicotine strength was 19.84 mg (SD 8.97, range 0–48 mg), reducing to an average 7.96 mg with their current device (SD 7.06, range 0–36 mg). The upper limit of nicotine in e-liquid in England is 20 mg/mL, and the maximum tank capacity 2 mL, as of May 2016, with a transition period until May 2017. The upper reported strength of 48 mg is beyond this limit, perhaps as they initiated vaping prior to transition or outside England, or due to errors in recall.

This study supports others suggesting vapers decrease their e-liquid nicotine strength over time [29–31], although those studies suggest users compensate for this by changing puffing patterns and using more e-liquid, maintaining cotinine levels. Research has shown that nicotine delivery is a function of device power, e-liquid nicotine concentration, and topography. Our study suggests participants may be transitioning from cig-a-likes with lower power and higher nicotine e-liquid concentrations to mod and tank devices with greater power and lower e-liquid nicotine concentrations. Whilst self-reported e-liquid nicotine concentration decreased, users may be taking in similar/more nicotine from these later-generation devices than the low power/high nicotine devices that have been shown to deliver nicotine poorly. We cannot tell from this study how nicotine intake changes over time.

This study reports novel findings suggesting starting on insufficient levels of nicotine based on previous CPD, combined with a less powerful device, may lead to greater risk of relapse. Sample size for that analysis was only 72, so further exploration of this hypothesis with a larger sample size, allowing for the inclusion of more confounders within the model, is warranted.

According to the 2017 ASH-A survey, among current users, fruit flavors were the most popular (28.5%), followed by tobacco (26.9%) and menthol/mint flavors (25.3%) [1]. Previous studies suggested fruit-flavored e-liquids are more popular among young people [31]. Our results suggest a change in flavor choices over time. We saw a significant reduction in use of tobacco flavor and increases in use of fruit/sweet/food flavors from initial to current flavor choice. An online survey by Russell et al. in the USA reported that initiating e-cigarette use with a tobacco-flavored e-liquid became less common between pre-2011 and 2015/16, and sweet flavors became more common [32]. Litt et al. report that among smokers asked to vape for 6 weeks, those given a menthol/tobacco flavor smoked less than those given cherry/chocolate [33]. Longitudinal surveys are required to see whether changing flavor over time, as well as flavor choice at initiation, might be beneficial to sustained smoking cessation. Preferences for fruit/sweet/food flavors may have implications for areas where flavors are banned [34].

### Limitations

This study is cross-sectional and so cannot identify causative associations. It relied on retrospective reports of vaping practices which may be subject to recall bias. Data on cessation were self-reported and not biochemically verified.

Another major limitation is missing data. Questions on flavors, device types, e-liquid nicotine concentration and vaping status were answered by more than 60% of participants, but questions on smoking status were answered by 56.9% of participants and questions on relapse, the main outcome variable, were only answered by 42% of participants. Many participants did not complete all questions, meaning some analyses are underpowered and some potential confounders could not be included in regression models. Pairwise deletion was used to manage missing data instead of multiple imputation, which may have been a more robust approach [35].

This survey recruited a convenience sample of e-cigarette users, likely representing those who were successful and wished to share their experiences. While not representative of the wider population of vapers, it does suggest ways in which smokers who have successfully switched to vaping may have achieved this successful transition.

Demographic characteristics of participants in this survey were compared with those of Smoking Toolkit Study participants who smoked cigarettes or any other tobacco product daily or occasionally at the time of the survey or during the preceding 12 months (Table 3) [36]. This suggested our sample were more likely to be of higher social grade and the percentage of female participants is much lower, which may be related to the perceived masculinity of vaping [22]. Mean reported CPD before cessation was 33.8 in this study, much higher than the 2015 UK average of 11.3 [36].

Weaver et al. highlight the variations in terminology (e.g., e-cigarette, vaping) and device descriptions (e.g. mod, personal vaporizer) among consumers and researchers [37]. This survey attempted to overcome these challenges by permitting participants to describe devices in their own words, but this was poorly completed, and the survey had to be adapted to a multiple-choice option.

None of the survey participants reported using Pod devices as these were not commonly used in the UK when the survey was conducted and so we are unable to comment on the potential role of these devices. Further research is needed into the effectiveness of these devices for sustained smoking cessation as their popularity grows.

### Future research

There is a need for studies on relapse to smoking among e-cigarette users that follow people up over time, providing data on trajectories, to understand how users experience progressing from simpler to more modern devices. If followed up over a long period, trends in e-cigarette use could be assessed, including whether people switching to e-cigarettes now or in the future are more likely to use a more modern device and so need a lower nicotine strength. Studies may consider how those switching from tobacco could be supported to choose a suitable device and strength, perhaps through working with vape shops and online retailers.

Future research could explore the potential role of flavors in relapse. Qualitative studies have suggested that perceiving e-cigarettes as something very different from tobacco smoking, rather than a substitute, is important for some vapers in avoiding a return to smoking [15]. The results of this study suggest a transition over time away from devices that look and feel like tobacco cigarettes, but further research is needed to investigate associations between e-liquid flavor and tobacco-smoking relapse.

## Conclusions

The results of this study suggest the choice of e-cigarette products and liquids used may have an impact on relapse to tobacco smoking. Those initiating vaping with a less sophisticated device and/or lower nicotine strength e-liquid may be at higher risk of relapse to tobacco smoking. Self-report patterns of device use by vapers suggest changing patterns over time, with many users moving from less sophisticated, tobacco-flavored cig-a-like devices, to more sophisticated tank devices with fruit/sweet/food flavors.

## Supplementary information

**Additional file 1:** Change in choice of flavour over time; demographic characteristics by device type; reasons for moving on from first device.

## Data Availability

The datasets used and/or analyzed during the current study are available from the corresponding author on reasonable request.

## Abbreviations

CPD: Cigarettes per day
NRT: Nicotine replacement therapy
OR: Odds ratio
RCT: Randomized controlled trial
UK: UK
USA: USA
