# Supplementary material for: Reported patterns of vaping to support long-term abstinence from smoking: a cross-sectional survey of a convenience sample of vapers
Source: Harm Reduct J. 2020 Oct 6;17:70. doi: 10.1186/s12954-020-00418-8 (PMC7541214; doi:10.1186/s12954-020-00418-8)
Supplement: Supplementary file 1 — Additional file 1: Change in choice of flavour over time; demographic characteristics by device type; reasons for moving on from first device. [file 12954_2020_418_MOESM1_ESM.docx]

Supplementary Material

**Table A1.** Change in choice of flavor over time.

| **Flavor** | **Initial (n)** | **Initial (%)** | **Final (n)** | **Final (%)** | **% Difference** | **95% CI for difference in proportions** |
| --- | --- | --- | --- | --- | --- | --- |
| Tobacco | 107 | 43.14 | 16 | 6.61 | -36.53 | -43.45 to -29.61 |
| Fruit, sweet or food | 69 | 27.80 | 144 | 59.50 | +31.7 | 23.34 to 39.97 |
| Various* | 58 | 23.39 | 72 | 29.75 | +6.36 | -1.44 to 14.16 |
| Menthol | 14 | 5.65 | 10 | 4.13 | -1.52 | -5.33 to 2.29 |
| Total | 248 | 100 | 242 | 100 |  |  |

*Note as this question was self-reported free text some participants recorded their flavour choice as ‘various’ with no further details provided. Those who reported using flavours from at least two of the included categories (tobacco, fruit/sweet/food and menthol) were also included in this classification.

**Table A2.** Demographic characteristics by device type.

| **Initial device type** | **% female (n=168)** | **Mean age (SD)** | **% SES (n=148)** | **Mean CPD** |
| --- | --- | --- | --- | --- |
| Cig-a-like | 22.5 | 50 (10.9) | AB 25.0% (n=13)  C 51.9% (n=27)  DE 23.1% (n=12) | 29 |
| Vape pen | 25.0 | 47 (11.3) | AB 14.1% (n=10)  C 45.1% (n=32)  DE 40.8% (n=29) | 29 |
| Mod and tank | 33.8 | 45 (12.9) | AB 12.0% (n=3)  C 60.0% (n=15)  DE 28.0% (n=7) | 29 |
| All | 23.6 | 49 (11.3) | AB 17.6% (n=26)  C 50.0% (n=74)  DE 32.4% (n=48) | 29 |

**Table A3.** Reasons for moving on from first device.

| **Reason** | **Participants (n=84)** |
| --- | --- |
| **Desire to upgrade** | **57** |
| Battery life/power | 12 |
| Flavor | 7 |
| Personalization / hobby / curiosity | 7 |
| Old technology | 2 |
| **Device inadequate** | **21** |
| Battery life/power | **8** |
| Flavor | **4** |
| Reliability | **1** |
| **Device no longer working** | **5** |
| **Nicotine strength too high** | **4** |
| **Initial device too expensive** | **2** |
